# The Management of Acute Adverse Effects of Breast Cancer Treatment in General Practice: A Video-Vignette Study

**DOI:** 10.2196/jmir.3585

**Published:** 2014-09-03

**Authors:** Moyez Jiwa, Anne Long, Tim Shaw, Georgina Pagey, Georgia Halkett, Vinita Pillai, Xingqiong Meng

**Affiliations:** ^1^Curtin UniversityPerthAustralia; ^2^Sydney Medical SchoolSydneyAustralia; ^3^Curtin UniveristyPerthAustralia

**Keywords:** breast cancer, treatment, general practice, adverse effects, patient care planning

## Abstract

**Background:**

There has been a focus recently on the use of the Internet and email to deliver education interventions to general practitioners (GPs). The treatment of breast cancer may include surgery, radiotherapy, chemotherapy, and/or hormone treatment. These treatments may have acute adverse effects. GPs need more information on the diagnosis and management of specific adverse effects encountered immediately after cancer treatment.

**Objective:**

The goal was to evaluate an Internet-based educational program developed for GPs to advise patients with acute adverse effects following breast cancer treatment.

**Methods:**

During phase 1, participants viewed 6 video vignettes of actor-patients reporting 1 of 6 acute symptoms following surgery and chemotherapy and/or radiotherapy treatment. GPs indicated their diagnosis and proposed management through an online survey program. They received feedback about each scenario in the form of a specialist clinic letter, as if the patient had been seen at a specialist clinic after they had attended the GP. This letter incorporated extracts from local guidelines on the management of the symptoms presented. This feedback was sent to the GPs electronically on the same survey platform. In phase 2, all GPs were invited to manage similar cases as phase 1. Their proposed management was compared to the guidelines. McNemar test was used to compare data from phases 1 and 2, and logistic regression was used to explore the GP characteristics that were associated with inappropriate case management.

**Results:**

A total of 50 GPs participated. Participants were younger and more likely to be female than other GPs in Australia. For 5 of 6 vignettes in phase 1, management was consistent with expert opinion in the minority of cases (6%-46%). Participant demographic characteristics had a variable effect on different management decisions in phase 1. The variables modeled explained 15%-28% of the differences observed. Diagnosis and management improved significantly in phase 2, especially for diarrhea, neutropenia, and seroma sample cases. The proportion of incorrect management responses was reduced to a minimum (25.3%-49.3%) in phase 2.

**Conclusions:**

There was evidence that providing feedback by experts on specific cases had an impact on GPs’ knowledge about how to appropriately manage acute treatment adverse effects. This educational intervention could be targeted to support the implementation of shared care during cancer treatment.

## Introduction

Breast cancer was the most common cancer in Australian women in 2009 (excluding nonmelanoma skin cancer) [1]. In Australia, 1 in 11 women will develop breast cancer in their lifetime [2]; 89% of women with breast cancer survive more than 5 years and die of unrelated causes [3]. The treatment of breast cancer may include surgery, radiotherapy, chemotherapy, and/or hormone treatment [4]. Treatment depends on prognosis, stage of disease, treatment options, and adverse effects, as well as the patient and her partner’s preferences [5].

Following adjuvant treatment, women may experience bowel disturbance, neutropenia, radiation dermatitis, and fatigue [6-7]. Posttreatment, brief follow-up is provided in the tertiary settings in some instances. Patients are also encouraged to see their general practitioner (GP) about any new or ongoing problems [8]. Previous studies have demonstrated that women consult a GP routinely in the months and years after treatment of breast cancer [9]. Physical adverse effects are a significant feature during breast cancer treatment [10]. Breast cancer patients are more likely to contact their GP for gastrointestinal symptoms than for other symptoms [11]. However, there is no evidence that these patients are advised correctly by GPs, and patients experience substantial unmet need for reassurance and advice [12]. There is also strong interest in the specialist sector to improve the support received by patients who have been treated for cancer [13]. To address the needs of patients treated for breast cancer, the GP needs to know how to effectively treat the adverse effects of therapy and understand the indications for urgent referral for specialist care. There is some evidence that GPs need more information on the diagnosis and management of specific adverse effects encountered immediately after cancer treatment [8].

There has been a recent focus on the use of the Internet and email to deliver education interventions to GPs. The use of video vignettes to explore medical decision making and to test other innovations is promising [14]. Preliminary evidence using video vignettes suggests that some innovations are not effective [15]. The value of letters from specialists about patients currently under the care of a GP is known to have an educational value; therefore, the study reported here incorporates this style of feedback education as an intervention delivered via the Internet in conjunction with video vignettes [16].

## Methods

### Overview

Following approval from the Curtin Human Research Ethics Committee, Perth, Western Australia (RD-68-12), participants were recruited from a previously established network of 150 GPs across Australia [15]. GPs were emailed invitations and nonresponders were followed up with personal invitations. Participants were remunerated with AUD $50 for their contribution.

### Materials

A total of 12 video vignettes were developed, 1 pair for each potential adverse effect related to treatment of breast cancer (see Textbox 1 for exemplars). Each vignette depicted a patient with clear indications for specific management, including referral, prescription, reassurance, and/or investigation [15]. The vignettes were developed by 3 GPs, a radiation oncologist, a medical oncologist, and a surgeon with reference to what they considered the most common complication immediately after treatment. The expert panel referenced best practice guidelines in the development of management options for each case with suggestions for prescription, referral for specialist treatment, and laboratory investigation. Each case had more than 1 correct management option (see Table 1).

**Details of patients presented in the video vignettes (a=phase 1, b=phase 2).**

Consultation 1a: “Marion Jones” 65-year-old female patient who had a lumpectomy 1 month ago. She is generally well. She suffers from hypertension and is treated with ramipril 5 mg. No allergies. Nonsmoker. She is now reporting a swelling at or near the surgical site with leakage of clear fluid through the lump. The area is not painful. Diagnosis: seroma

Consultation 1b: “Anne O’Brien” 60-year-old female patient had a left mastectomy and lymph node dissection 10 weeks ago. Now presents with a persistent painless swelling in her left axilla. She has been treated for depression with fluoxetine 20 mg/day and takes tamoxifen. Her husband is very worried about this lump and feels the cancer may be back. On examination, this is a soft fluctuant mass with mild erythema above the lesion. Diagnosis: seroma

Consultation 2a: “Christine Wilkins”, 50-year-old female patient had chemotherapy for breast cancer 4 days ago. Now presents with lethargy, moderate sore throat, and fever >38 °C. On examination, her throat is inflamed. She has no past medical history of note. She has no rigors or chills. She is allergic to penicillin and cephalosporins. Diagnosis: postchemotherapy infection (possible neutropenia)

Consultation 2b: “Marilyn Michaels” 56-year-old female patient had chemotherapy for breast cancer 9 days ago. Now presents with lethargy, slight dysuria, and fever >38 °C, BP <100 systolic, pulse >100. On examination, her urine is clear—no hematuria or proteinuria. She has no past medical history of note. She has no rigors or chills. Diagnosis: postchemotherapy infection (possible neutropenia)

Consultation 3a: “Margaret Enright” 59-year-old female patient had chemotherapy for breast cancer 9 days ago. Now presents with diarrhea for past 3 days. She has no abdominal pain and there is no blood in the motions. However, she has an episode of diarrhea every 2 hours and she feels weak and tired. She is not taking any medication and is allergic to penicillin. She lives with her sister who is working in the city and not able to be with her all day. On examination, she looks pale and tired. Her blood pressure is normal. Her abdomen is soft, nontender. Diagnosis: postchemotherapy diarrhea

Consultation 3b: “Francis Burn” 50-year-old female patient had chemotherapy for breast cancer 6 days ago. She has had 3 episodes of loose, watery bowel movements every day for the past 3 days. Her partner is concerned because her appetite is reduced and she has been feeling weak and tired. On examination, she is apyrexial but looks pale and tired. Her abdomen is soft, nontender. She is not clinically dehydrated, her blood pressure is normal. Diagnosis: postchemotherapy diarrhea

Consultation 4a: “Michelle Sands” 70-year-old female patient had chemotherapy for breast cancer 2 days ago now presents with vomiting 10 times a day. She has mild abdominal pain and feels thirsty but has lost her appetite. She denies any chest or abdominal pain. She is tired because she says she has not slept very well. Her son, who lives a few minutes away, is worried about her. She takes metformin 500 mg 3 times daily and amlodipine 5 mg/day. Diagnosis: postchemotherapy vomiting

Consultation 4b: “Sandra Speers” 55-year-old female patient had chemotherapy for breast cancer 4 days ago now presents with vomiting 4 times a day. She has mild lower abdominal pain and feels thirsty but has lost her appetite. She is tired because she has not slept very well. Her daughter is worried about her. She has had a myocardial infarction 6 months ago and is maintained on atenolol 50 mg/day and amlodipine 10 mg/day. She denies chest pain or breathlessness. On examination, there are no clinical signs of abdominal pathology. Diagnosis: postchemotherapy vomiting

Consultation 5a: “Susan Smith” 60-year-old female patient has been receiving full left breast radiation therapy for 5 weeks. She presents with a painful, itchy erythema and patchy, moist desquamation. She is aware that this is probably an adverse effect of the radiotherapy and wonders if anything can be done to make her more comfortable. She is also concerned that the therapy will scar her skin. She wonders if she is still fit to receive more radiotherapy. She is also worried if the therapy is having a bad effect on her ribs, heart, and lungs. Diagnosis: radiation dermatitis

Consultation 5b: “Doris Daniels” 50-year-old female is receiving radiotherapy to the right breast and axilla. She now presents with painful, itchy erythema and patchy, moist desquamation, especially in the skin folds. Paracetamol does not help the pain. She can’t wear a brassiere. She has been using topical propylene glycol but doesn’t find it particularly helpful. She is going to her sister’s wedding in a couple of days and would like to know if anything can help her be more comfortable. Diagnosis: radiation mastitis

Consultation 6a: “Alex Horner” 40-year-old female is receiving adjuvant chemotherapy for breast cancer. She now presents with constipation of 3 days duration. She has had very minimal bowel movements, mostly mucous and flatulence. She feels bloated and uncomfortable. On examination, she is bloated and there are minimal bowel sounds. On rectal examination, she is impacted with hard feces. Diagnosis: postchemotherapy constipation

Consultation 6b: “Michelle Marshall” 43-year-old female receiving chemotherapy for adjuvant breast cancer. She now complains of generalized colicky abdominal pain and constipation of 6 days duration. She has had no bowel movements in that time. She is very uncomfortable and occasionally troubled by the pain. She has had minimal oral intake and appears dehydrated. On examination, she is bloated and slightly tender throughout her abdomen. On rectal examination, she is impacted with feces and bleeding slightly from a hemorrhoid. Diagnosis: postchemotherapy constipation

**Table 1:** Specific recommendations for management of symptoms or problems after treatment for breast cancer.

Symptom or problem		Adverse effect		
		Mild	Moderate	Severe
**Seroma**				
	Characterized by	Slight fluctuant swelling in operative site	Obvious fluctuant swelling in operative site	Tense (nonfluctuant) and uncomfortable swelling in operative site
	Action to be taken by GP	Arrange for patient to see surgeon within 7 days	Arrange for patient to see surgeon within 4 days	Arrange for patient to see surgeon within 2 to 3 d
**Infection**		All the following:	Any of the following:	Any of the following:
	Characterized by	Fever <38 °C Does not feel unwell BP normal Pulse <100	Fever >38 °C Mild symptoms plus BP <100 systolic Pulse >100	Fever >38 °C Symptoms including: Rigors/chills Dizziness BP <100 systolic Pulse >100
	Action if <7 d or >14 d since chemotherapy	See patient <48 h Paracetamol regularly Review 1-2 d if worse	See patient <24 h Paracetamol regularly Oral antibiotics	Call medical oncologist. Send immediately to closest emergency department (ED). If patient from rural area and closest hospital >2 h away, consider giving IV/IM broad spectrum antibiotic prior to sending
	Action if 7-14 d since chemotherapy	See patient <24 h Paracetamol regularly Oral antibiotics Review 1 d, if worse call medical oncologist	Call medical oncologist	Call medical oncologist Send immediately to closest ED. If patient from rural area and closest hospital >2 h away consider giving IV/IM broad spectrum antibiotic prior to sending
**Diarrhea**		≤4/d	5-8/d	>8/d±abdominal pain or dehydrated and cannot cope at home
	Action to be taken	See patient <48 h Imodium after every bowel movement (BM) up to 8/d. If not controlled on this, then to return for review sooner and to trial codeine	See patient <24 h Trial of loperamide (2 after first BM and 1 after each subsequent), if insufficient then codeine phosphate 30 mg after each BM max 4/d	Call medical oncologist
**Vomiting**				
	Characterized by	≤ 6/d still managing oral intake	7-10/d with mild oral intake	>10/d no oral intake
	Action to be taken	See patient <48 h prochlorperazine maleate suppositories ± Ondansetron wafer 8 mg bid	See patient < 24 h prochlorperazine maleate suppositories ± Ondansetron 8 mg bid Call Medical Oncology Consider admission	Call medical oncologist and consider admission
**Radiation dermatitis**		All of the following	Any of the following	Any of the following
	Characterized by	Faint skin erythema ± dry desquamation ± moderate pruritus	Brisk erythema ± patchy moist desquamation limited to skin folds/creases	Confluent moist desquamation ± erythema extending beyond treatment area ± systemically unwell
	Action to be taken	Avoid additional skin irritants (eg, sun exposure, perfumed soaps/perfumes, adhesive tapes) Wash area daily in warm/tepid water Moisturize daily-tid with water-soluble moisturizer Simple analgesics Consider sparing use of topical steroids for patchy pruritus as long as there is no evidence of desquamation or skin breakdown See patient <48 h re: compliance	As above re: avoidance of skin irritants, simple hygiene, measures and analgesics Daily or twice daily nonadherent dressings for comfort See patient daily until resolution Contact Radiotherapy Department <48 h for advice/dressings	Analgesia as appropriate Daily or twice daily nonadherent dressings Commence empirical systemic antibiotics—only if indicated by evidence of systemic infection, recommend radiation oncologist review Contact radiation oncologist/Radiotherapy Department <24 h
**Constipation**				
	Characterized by	≤2 d of nil/minimal BM	3-5 d of nil/minimal BM	>5 d of nil/minimal BM ± colic
	Action to be taken	Recommend docusate sodium & senna II bid and or macrogol 3350 sachet I bid and glycerine suppository See patient <48 h	Increase docusate sodium and senna and macrogol 3350 consider addition of sorbitol etc or Macrogol AND sodium citrate suppositories See patient <24 h	Call medical oncologist Consider saline enema

The vignettes were then prepared as a short video monolog by an actor-patient (see Multimedia Appendix 1). The video included an off-camera commentary by an actor-doctor describing relevant signs to be found on clinical examination. Each of the 6 pairs was then randomly assigned to phase 1 or phase 2 of the study. Participation in the study was via the Internet. Participants were asked four questions after watching each video vignette:

1. What is your diagnosis?
2. Would you prescribe something? If so, what?
3. Would you refer the patient? If so, to whom?
4. Would you order tests? If so, which tests?

The responses were recorded via the Internet platform used to administer the survey. After responding to the first 6 of 12 videos, participants were provided written feedback in the style of a letter from a specialist clinic, highlighting the recommended guidelines for managing the adverse effect in each case.

The project was completed in two phases. In phase 1, participants were invited to view the first set of 6 videos and describe their management of the standardized patient depicted. All participants received expert feedback on the management of the cases viewed within 2 weeks of the project coordinator receiving their proposed management plan. The feedback was in the form of a letter written as if the patient had attended a specialist clinic immediately after consulting the GP. The letters did not refer to the GPs proposed plan for the patient, but stated “For Marion Jones [Consultation 1a], I would recommend the following...”. The letter also outlined the protocol for the general management of her symptoms if they were mild, moderate, or severe. In most cases, there was more than 1 action that the GP could have taken to manage the case as per specialist guidelines. The letters were sent via the Internet using the same Qualtrics survey platform. Once the study coordinator was alerted by the system that the participant had opened the letter for each case, they were sent a link to phase 2. In phase 2, all participating GPs were invited to view the second set of 6 videos and to describe their management of the standardized patient depicted. The phase 2 vignettes matched those in phase 1 by diagnosis (see Textbox 1).

### Statistical Analysis

We hypothesized that the proportion of those who managed cases as per the expert recommendations would be greater after feedback (60% vs 30%). Therefore, a sample of 42 participants per group was deemed sufficient in this exploratory study [17].

The McNemar test was used to determine phase differences in the proportion of cases diagnosed and managed correctly. The phase 1 data offered the opportunity to investigate the GP characteristics that were associated with an incorrect response. GPs’ characteristics associated with inappropriate case management were explored by using logistic regression models using phase 1 data. This helped to identify which groups of practitioners might best be targeted for the intervention and may be different for each of the case types. A full regression model included the following variables: age, sex, country of graduation, years after graduation, years of GP experience, status as established GP or GP registrar (trainee general practitioner), recognized speciality qualification with the Royal Australian College of General Practitioners (Fellow of the Royal Australian College of General Practitioners, FRACGP), the remoteness of their primary practice, the number of GPs at their primary practice, status as a principal (practice owner) within their primary practice, number of patients seen per week, total patient care hours per week, and whether they conducted consultations in languages other than English. Regression models were constructed using both backwards elimination and forward selection. Univariate modeling was performed before the stepwise regressions, and the results were used to guide the reduction of the full models. Variables with a *P* value less than .05 were retained in the final model and reported. Stata version 12.1 (StataCorp LP, College Station, TX, USA) was used to perform the analysis. Logistic regression models were adjusted for the lack of independence between individual participants by estimating the clustered standard errors to account for intragroup correlation (vce option in Stata). No special technique was used to handle the missing values because there are very few missing values in this study. For participants’ demographics, only the variable “sessions per week” had 2 missing values (4% of total), and there was no missing value for the outcome variables.

## Results

A total of 50 GPs consented to participate and completed the study. GPs self-reported their demographic characteristics (see Table 2). Those who participated in the study were younger than Australian GPs generally (mean age 43.4 years vs 50.5 years) and a greater proportion were female (52% vs 39.1%), registrars (18% vs 3.8%), and Australian graduates (76% vs 65.9%) [18-20]. Most participants (62%-100%) correctly diagnosed cases in this study, especially in phase 2 (see Table 3). However, there were significant differences in the management of cases between the two phases and between cases (see Table 4).

**Table 2:** Participant demographic information (N=50).

GP characteristics			Study sample	National population^a^	
				Mean	%
**Demographics**					
	Age (years), mean (SD)		43.4 (11.0)	50.5	
	Years after graduation, mean (SD)		19.5 (11.2)		
	Years of GP experience, mean (SD)		14.8 (11.3)		
	GPs at primary clinic, mean (SD)		7.7 (4.0)		
	GP sessions worked/week (n=48), mean (SD)		6.5 (3.1)		
	**Sex, n (%)**				
		Male	24 (48)		60.9
		Female	26 (52)		
	Graduated in Australia, n (%)		38 (76)		65.9
	Registrars (GPs in training), n (%)		9 (18)		3.8
	FRACGP (Fellows of the Royal Australian College of GPs), n (%)		30 (60)		56.8
**Practice demographics**					
	Accredited, n (%)		49 (98)		88.6
	**Location (Australian state/territory), n (%)**				
		Australian Capital Territory	1 (2)		1.5
		New South Wales	7 (14)		33.1
		Queensland	3 (6)		19.5
		South Australia	6 (12)		8.4
		Victoria	13 (26)		25.1
		Western Australia	20 (40)		9.1
	**Clinic remoteness, n (%)**				
		Major city	38 (76)		71.1
		Nonmajor city	12 (24)		28.9
	**GP position, n (%)**				
		Principal	9 (18)		
		Nonprincipal	32 (64)		
		Others	9 (18)		
**Patient consultations**					
	**Patient consultations per week, n (%)**				
		<100	27 (54)		
		100-149	14 (28)		
		≥150	9 (18)		
	**Patient consultations hours per week, n (%)**				
		<11	7 (14)		1.2
		11-20	8 (16)		12.2
		21-40	27 (54)		53
		≥41	8 (16)		33.5
	**Non-English consultations, n (%)**				
		No	43 (86)		
		Yes, <25%	7 (14)		24.5

^a^Sourced from national data when available [18-20].

**Table 3:** Correct diagnosis of cases per phase of study (N=50).

Diagnosis	Phase 1, n (%) (n=50)	Phase 2, n (%) (n=50)	*P* value^a^
Constipation	40 (80)	39 (78)	.80
Diarrhea	46 (92)	50 (100)	.046
Radiation dermatitis	47 (94)	47 (94)	>0.99
Postchemotherapy infection	44 (88)	49 (98)	.03
Postoperative seroma	31 (62)	49 (98)	<.001
Vomiting	47 (94)	50 (100)	.08
Total (n=300)	255 (85.0)	284 (94.7)	<.001

^a^ *P* values derived from McNemar test.

**Table 4:** Correct management of cases by phase of study (N=50).^a^

Management		Phase 1, n (%) (n=50)	Phase 2, n (%) (n=50)	*P* value^b^
**Constipation**				
	Refer to oncologist	3 (6)	8 (16)	.14
	Prescribe medication	38 (76)	44 (88)	.11
**Diarrhea**				
	Prescribe Medication	32 (64)	45 (90)	.003
**Radiation dermatitis**				
	Refer to breast care nurse / radiation oncologist / specialist	33 (66)	25 (50)	.09
	Prescribe specific creams	3 (6)	19 (38)	<.001
	Advise patient	13 (26)	38 (76)	<.001
**Postchemotherapy infection**				
	Refer to oncologist	18 (36)	27 (54)	.07
	Collect throat swab	18 (36)	33 (66)	.003
**Postoperation seroma**				
	Refer to surgeon	23 (46)	40 (80)	.001
	Organize ultrasound	35 (70)	39 (78)	.32
	Aspiration	16 (32)	13 (26)	.49
**Vomiting**				
	Refer to oncologist	20 (40)	5 (10)	<.001
	Prescribe medication	22 (44)	46 (92)	<.001
	Order tests	20 (40)	4 (8)	<.001

^a^In each case there is more than 1 correct answer.

^b^ *P* values derived from McNemar test.

Regression analysis was carried out to determine the variables associated with management of adverse effects that were not consistent with expert opinion in phase 1. GPs failed to manage the cases as recommended by experts with reference to 2 explanatory variables:

1. GP demographics
2. Individual vignettes

In phase 1, as shown in Table 5, Australian graduates were statistically less likely to offer an inappropriate referral. Male GPs and participants who identified themselves as neither practice owner nor employee (most likely locum practitioners) were less likely to provide an inappropriate prescription. GPs who worked more sessions per week were more likely to offer an inappropriate prescription. Older GPs were less likely to order a unnecessary test. In contrast, those who consulted for more than 20 hours per week were more likely to order an unnecessary test. Compared to managing constipation or diarrhea, participants were less likely to make an inappropriate referral. Patients with radiation dermatitis, postchemotherapy infection, or vomiting were more likely to be given an inappropriate prescription and test.

These variables explained 15%-28% of the differences observed (Pseudo *R* ^*2*^=15%, 28%, and 15% for inappropriate referral, prescription, and test order, respectively) (see Table 5). Results in Table 5 are odds ratios and 95% confidence intervals derived from 3 logistic regression models adjusted for clustering of GPs. Only variables with *P* values <.05 were included in the final model and reported in Table 5.

Overall, all three aspects of management had improved significantly in phase 2 as shown in Table 6.

**Table 5:** Factors associated with incorrect management (inconsistent with expert opinion) in phase 1 (N=50).

Explanatory variable		Inappropriate referral		Inappropriate prescription		Unnecessary tests	
		OR (95% CI)	*P*	OR (95% CI)	*P*	OR (95% CI)	*P*
Age		—		—		0.98 (0.96, 1.00)	.04
GP sessions worked/week		—		1.12 (1.03, 1.22)	.008	—	
**Sex**		—				—	
	Female			1.00			
	Male			0.44 (0.26, 0.74)	.002		
**Graduated in Australia**				—		—	
	No	1.00					
	Yes	0.47 (0.23, 0.97)	.04				
**Patient consultations hours/week**		—		—			
	<21					1.00	
	21-40					1.87 (1.08, 3.22)	.03
	>41					2.01 (1.14, 3.56)	.02
**Position**		—				—	
	Principal			1.00			
	Nonprincipal			0.49 (0.21, 1.11)	.09		
	Others			0.25 (0.09, 0.71)	.009		
**Cases**							
	Constipation	1.00		1.00		1.00	
	Diarrhea	0.29 (0.07, 1.25)	.10	1.90 (0.78, 4.62)	.16	15.92 (6.11, 41.47)	<.001
	Radiation dermatitis	0.03 (0.01, 0.11)	<.001	59.41 (14.05, 251.26)	<.001	2.19 (0.88, 5.46)	.09
	Postchemotherapy infection	0.11 (0.03, 0.38)	<.001	4.66 (1.82, 11.91)	.001	3.15 (1.23, 8.07)	.02
	Postoperative seroma	0.07 (0.02, 0.30)	<.001	0, 30 (0.08, 1.04)	.06	1.62 (0.53, 5.00)	.40
	Vomiting	0.09 (0.03, 0.32)	<.001	4.66 (1.90, 11.43)	.001	7.32 (2.49, 21.59)	<.001

**Table 6:** Incorrect management by phase.

Management	Phase 1, n (%) (n=300)	Phase 2, n (%) (n=300)	*P* value^a^
Refer to specialist	194 (64.7)	148 (49.3)	<.001
Prescription	138 (46.0)	76 (25.3)	<.001
Order test	126 (42.0)	103 (34.3)	.03

^a^ *P* values derived from McNemar test.

## Discussion

These data indicate that although most participants correctly diagnosed the conditions presented throughout the study, limited numbers knew how to manage the acute adverse effects of breast cancer treatment. Australian graduates performed better, but those who worked longer hours were more likely to make questionable decisions in this study. The latter may reflect research that longer hours have a negative impact on job performance [21]. This study did not test performance with real patients or in conditions of varying levels of fatigue; therefore, the comments remain speculative. We also note that practitioners who worked longer hours were more likely to order unnecessary tests. It is possible that this group is more comfortable with trying to manage cases on their own rather than refer back to an oncologist. However, we were unable to explore this hypothesis with the data collected.

The management of radiation dermatitis, postchemotherapy infection, and vomiting proved the most challenging. For almost every case, the management improved following feedback. These differences were marked for seroma, postchemotherapy infection, and diarrhea. This is an important observation which suggests that, if this study had been conducted with real patients, there was scope for significant harm because of diagnostic or management failures. Participants were more likely to diagnose and refer a seroma after feedback. Such differences in the management of acute adverse effects by GPs have not been reported previously because most patients are likely to consult their specialist within days or weeks of treatment rather than a GP [22].

Some adverse effects, such as persistent vomiting after chemotherapy, are likely to be emergencies; others, such as seromas, are distressing to patients, but unlikely to be life threatening. Some adverse effects, such as a postchemotherapy infection, can cause significant harm if they go unrecognized [23]. It has been suggested that GPs should play a much more active part during the treatment phase of the patient’s cancer journey [24]. If this is to be the case, then GPs need to be trained to manage the common acute effects of cancer treatment and at the very least these conditions need to feature in the differential diagnosis of patients presenting with symptoms soon after treatment of breast cancer [6,7].

Differences in the proposed management between the participants and the expert panel were less marked in phase 2 (after feedback). Such improvements, if they were noted in actual clinical practice, would lead to a reduction in adverse incidents, and better outcomes and satisfaction for patients. For example, as shown in Table 4, in phase 1 only 6% of participants prescribed the appropriate treatment of radiation dermatitis, whereas in phase 2 this proportion increased to 38%. In the case of the possible neutropenia, a significant proportion would arrange appropriate investigations in phase 2. This increases the potential for shared care between health sectors and makes it more likely treatment would be offered sooner rather than later. This is especially the case where patients may suffer avoidable harm if the practitioner in the community is able to recognize the need for urgent specialist advice for someone receiving lifesaving treatment. There were still significant numbers of participants whose proposed management of the vignettes was not consistent with expert opinion. In this study, it was not clear whether this was because participants disagreed with the suggested treatment plans or failed to assimilate the feedback into the phase 2 responses. Although there was a marked improvement in the management of cases, it would be unsafe to assume this was entirely related to the feedback received after phase 1. In the case of chemotherapy-induced vomiting in phase 2, although the participants were more likely to prescribe an antiemetic, they were less likely to refer back to the oncologist or to order the relevant tests after feedback. This was unexpected. It is possible that the scenario presented was considered “mild” because the patient was reported to have vomited only 4 times a day, in which case it may have been deemed unnecessary to refer to the oncologist or arrange laboratory tests to check the renal function. A future study involving this scenario would need to make it clearer that the patient was in need of specialist advice. Therefore, more severe symptoms would need to be presented in the vignette.

A recent literature review reported that two other factors are also likely to be important in the context of a cancer diagnosis: attitudes and beliefs [24]. These issues were not evaluated in this study. For example, we were unable to report the participants’ attitudes toward the management of patients with acute adverse effects and whether they felt this role extended to investigating and treating acute conditions that may have resulted from specialist treatment [25]. A diversity of opinions in regards to this issue have been described among Australian GPs in previous reviews [26]. Nor could we confirm that all participants had easy access to the relevant specialists and/or would have had the option to refer a patient urgently with a condition they had not previously encountered to such an expert. The available evidence suggests that this is not a safe assumption and that management plans would be impacted by the clinicians’ experience in their local context [13].

This pilot study had a modest sample size, which was estimated based on the hypothesis that the participants would be twice as likely to manage patients as per expert opinion following feedback on a similar previous case. This was not true of all management modalities. In some cases, any significant improvement in phase 2, as shown in Table 4, was much more modest. Therefore, a much larger study would be required to robustly demonstrate that this mode of education is likely to increase GPs’ knowledge. Because no other educational intervention was offered in a randomized experimental design, the conclusions that can be drawn from these data are also limited.

This study with vignette-based feedback showed promising results that managing the common adverse effects of cancer treatment could be delegated to general practice. Such an intervention could support the application of shared care models of care. A larger study, including management of adverse effects in real patients, needs to be conducted before it can be safely recommended. However, noting that some patients with potentially life-threatening adverse effects may not be managed appropriately suggests a need for safeguards to protect patients in a study with bona fide patients.

## Multimedia Appendix 1

Example of video vignette.

## Abbreviations

ED: emergency department
GP: general practitioner
BM: bowel movement
